# Hope for the best prepare for the worst: acute kidney disease and catastrophic comorbidities (a case report)

**DOI:** 10.11604/pamj.2024.49.134.45331

**Published:** 2024-12-27

**Authors:** Kübra Kaynar, Elif Çağlayan, Nil Bahar Atasoy, Betül Kekeç, Murat Küçükbirinci, Muhammed Bilal Bektaşoğlu

**Affiliations:** 1 Department of Nephrology, Faculty of Medicine, Karadeniz Technical University, Trabzon, Turkey,; 2 Department of Internal Medicine, Faculty of Medicine, Karadeniz Technical University, Trabzon, Turkey

**Keywords:** Acute kidney injury, dialysis, recovery of function, case report

## Abstract

It is evident that Acute Kidney Injury (AKI) is an independent risk factor for both the survival of patients and their kidneys. Here, we present a case of oliguric AKI secondary to blunt trauma-induced crush syndrome complicated with severe sepsis in which the patient had a complete recovery of kidney functions after 3 months of dialysis dependency. A 40-year-old male construction worker had a severe episode of work accident. He had fallen into the stream from a height of 6 meters and the concrete pillars of the bridge fell over him. He had an iliac artery injury, crush-related acute kidney failure, a ruptured bladder, multiple fractures in the lumbar vertebral spinous and transverse processes, bilateral pelvic rami, acetabulum, and bilateral iliac wings. Despite 3 months of dialysis dependency with multiple episodes of sepsis and nephrotoxic antibiotic applications, fortunately, recovery of kidney functions (creatinine clearance >20 mL/min) was achieved. The creatinine clearance of the patient was calculated as 78 mL/min one year after discharge from the hospital. It is well-known that severe trauma leading to severe sepsis and severe AKI has catastrophic effects on the survival of patients. In addition, nephrotoxic antibiotics and contrast media had to be given to our patient, which resulted in further injury. A multidisciplinary (including nursery care) approach, early and proper treatment of sepsis, pulmonary rehabilitation, enteral/parenteral nutritional support, and appropriate timing, prescription, and dose of dialysis are fundamental factors playing a major role in the recovery of prolonged AKI as in our patient.

## Introduction

It is well known that acute kidney injury (AKI) is an independent risk factor for both the survival of patients and their kidneys. Thus, one of the complications of AKI is the development of End-Stage Kidney Disease (ESKD). When the need for Kidney Replacement Therapy (KRT) after AKI development lasts more than 3 months, Kidney Disease Improving Global Outcome (KDIGO) guidelines recommend evaluation for new-onset ESKD complications after AKI [1]. Recently, it has been defined as AKI, Acute Kidney Disease (AKD), and Chronic Kidney Disease (CKD) when the duration of impaired kidney functions is ≤ 7 days, 8 days to ≤ 3 months, and > 3 months, respectively [2]. However, the strength of this recommendation has not been graded [1]. Thus, close and careful monitoring may improve kidney functions even after more than 3 months of dialysis dependency of patients with AKD. A study from mayo clinic reported that 22 patients were recovered out of 42 AKI patients with normal baseline kidney functions after 3 months of hemodialysis dependency. However, the authors did not mention the ratio of patients with complete renal recovery [3]. In addition, 52 other patients from different centers with delayed and sufficient (not complete) recovery of kidney functions after a range of 3.5 to 69 months of dialysis dependency were also presented [4]. Here, we present a case of oliguric AKD secondary to blunt trauma-induced crush syndrome complicated with sepsis in which the patient had complete recovery of kidney functions after 3 months of KRT. The complete recovery of repetitive kidney injury with catastrophic comorbidities is very rarely reported in the literature, even though it is known that severely and rapidly diseased kidneys could be recovered in up to three months.

## Patient and observation

**Figure 1:**
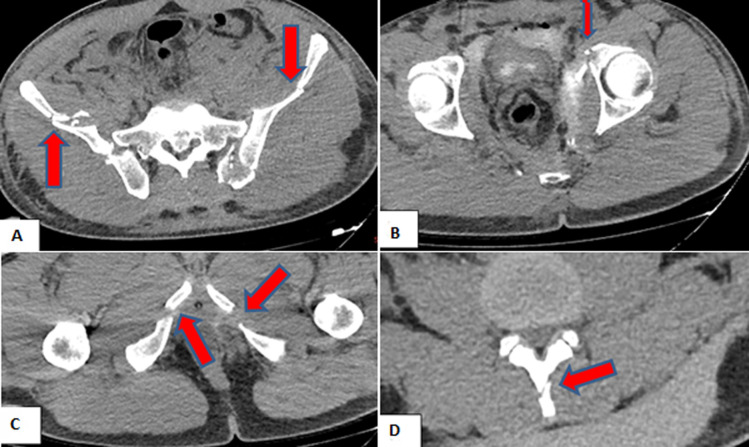
axial plain images of computed tomography showing: A) multiple iliac wings fractures; B) left acetabular fracture; C) fractures of bilateral pubic rami; D) fracture in the lumbar vertebral spinous process (L3) (red arrows)

**Patient information:** a 40-year-old male construction worker with healthy family medical history and without past history of intervention had a severe episode of work accident on April 1, 2022. He had fallen into the stream from a height of 6 meters and the concrete pillars of the bridge fell over him. The patient without any previous history of medical and familial comorbidity, was admitted to our center with iliac artery injury, crush-related acute kidney failure, multiple fractures in the lumbar (L1-L5) vertebral spinous and transverse processes, bilateral pelvic rami, acetabulum, and bilateral iliac wings (Figure 1).

**Clinical findings:** his consciousness and vital signs were normal at admission. He initially had suprapubic tenderness, scrotal ecchymosis, and reddish-black urine. Intense fluid resuscitation resulted in the clarification of urine color.

**Figure 2:**
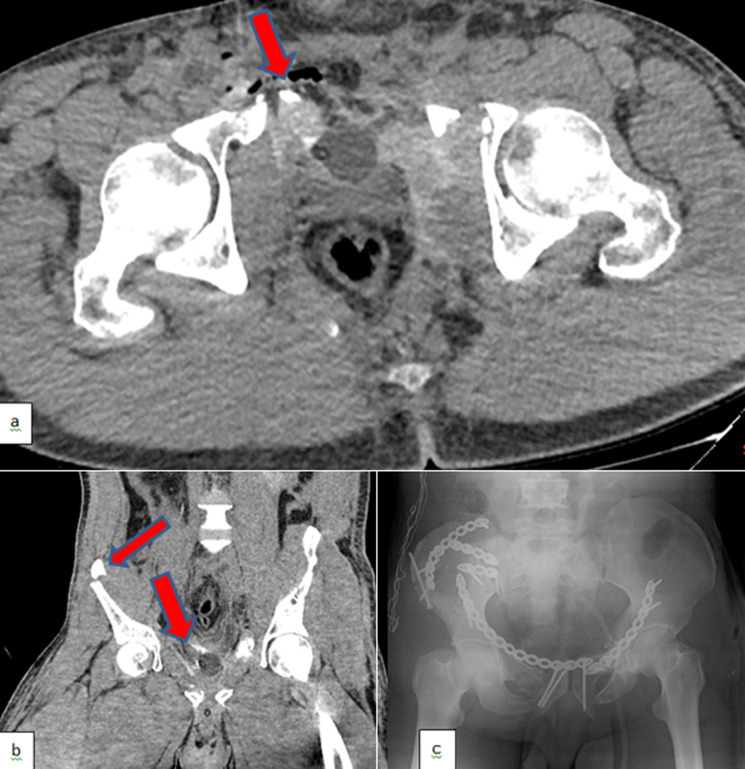
a) axial plain images of computed tomography showing free air and extravasation of contrast from the bladder; b) coronal plain images of computed tomography showing iliac wing fracture and extravasation of contrast from the bladder; c) plain X-ray showing multiple screw fixations and surgical plates (red arrows)

**Diagnostic assessment:** however, Computed Tomography (CT) revealed the perforation of the bladder from the neck to the dome (Figure 2). The Injury Severity Score (ISS) of the patient was 30. His urine output, serum myoglobin levels, and kidney functions mildly improved after fluid resuscitation (Table 1). However, his urine output relatively decreased on the third day, tachypnea, fever, and tachycardia developed, and oliguria was detected on the fifth day of admission due to sepsis. There were no diagnostic challenges.

**Table 1:** initial course of the biochemical and clinical abnormalities associated with rhabdomyolysis-related acute kidney failure

Parameters	At admission	Next day	On second day	On third day	On fourth day
Serum creatinine level (mg/dL)	4.54	4.96	1.98	2.23	4.84
Urine volume (mL/day)	2000	8050	6900	3610	460
Serum myoglobin level (mg/L)	52852	15260	8455	6731	9977
Alanine aminotransferase (ALT) U/L	123	117	133	171	467
Serum creatine kinase level (U/L)	46300	47811	48453	41689	43831
Lactate dehydrogenase (LDH) U/L	1200	1574	2008	2243	3113
Serum potassium level (meq/L)	6.6	5.9	3,3	3.3	3,7
Body temperature (°C)	36	37.5	37.4	37,8	39
C-reactive protein (mg/L)	242	425	577	-	687

u/l: ug/l

**Diagnosis:** he was transferred to the intensive care unit with polytrauma, multiple fractures, rupture of bladder, sepsis, and rhabdomyolysis-related AKI with a serum creatinine level of 4.54 mg/dL and a myoglobin level of 52852 µg/L (Table 1).

**Figure 3:**
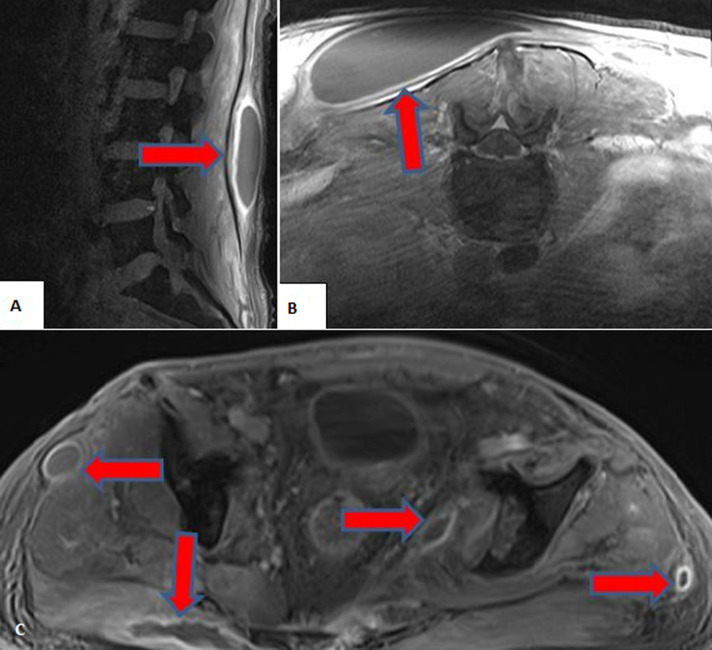
A) magnetic resonance imaging sections displaying; B) subcutaneous abscess in the left lumbar area; C) multiple abscesses (red arrows)

**Therapeutic interventions:** he was intubated, and continuous veno-venous hemodialysis (CVVHD) was initiated due to hypervolemia. Parenteral, enteral nutritional support and pulmonary rehabilitation were applied in the meantime. The urologists repaired the ruptured bladder, and the orthopedists fixated multiple fractures of the bones with screws and surgical plates when the patient became hemodynamically stable, under treatment of multiple antibiotics and vasoactive medications (Figure 2). He was extubated after ten days of follow-up, and CVVHD was replaced by intermittent hemodialysis (HD). The frequency of HD (weekly kt/V of 3.9) was performed according to the recommendations of the 2012 Kidney Disease Improving Global Outcomes (KDIGO) guidelines [5]. Unfortunately, he had several episodes of sepsis due to central venous catheter infections (*Staphylococcus epidermidis, Staphylococcus haemolyticus*, methicillin-resistant *Staphylococcus aureus*, and *Pseudomonas aeruginosa were detected in cultures*), soft tissue abscesses and infections (*Candida parapsilosis*), and urinary tract infection (*Citrobacter freundii*). Several nephrotoxic antibiotics such as tigecycline, colistin, and teicoplanin had to be given to the patient due to the resistance of the pathogens (Figure 3).

No adverse reactions were noticed. Central venous catheters were replaced eight times due to these infections. He also had diarrhea, the etiology of which was considered to be enteral feeding formulas and antibiotics. Control magnetic resonance imaging revealed multiple abscesses, the drainage of which was performed (Figure 3). The patient was followed up in an intensive care unit for one month. After hemodynamic stabilization was achieved, he was admitted to the nephrology department and followed up for another two months. A kidney biopsy was not performed because unexplained acute kidney injury and glomerular proteinuria were not present. The patient had microalbuminuria (albuminuria=253 mg/day) and tubular proteinuria (beta 2 microglobulinemia= 7390 µg/L). Antinuclear antibody (ANA), anti-double-stranded deoxyribonucleic acid (anti-dsDNA) antibodies, anti-ENA -extractable nuclear antigens- antibodies, and anti-neutrophil cytoplasmic antibodies (ANCA) were not detected. After two months of an oliguric period with multiple episodes of sepsis and nephrotoxic antibiotic applications, an arteriovenous fistula (AVF) was created considering a complete and irreversible loss of kidney functions. An increase in urine output was noticed in the following days, although creatinine clearance was inadequate (<12 mL/min) to allow discontinuation of HD.

**Follow up and outcome of interventions:** recovery of kidney functions (creatinine clearance >20 mL/min) was detected after 3 months of dialysis dependency. The creatinine clearance of the patient was calculated as 78 mL/min and 94 mL/min at 12 months and 18 months after discharge respectively. Albuminuria was 18 mg/day at 18 months after discharge. The closure of the AVF was recommended.

**Patient perspective:** he was very pleased to be free of dialysis, after all those days of suffering, intensive treatments and dialysis.

**Informed consent:** written informed consent for publication of his medical report, and for all the interventions were obtained from the patient.

## Discussion

Acute kidney injury, which is defined as a sudden loss of kidney functions has the risks of increased mortality, cardiovascular events, and transition to CKD. Generally, acute kidney injury is caused by extrarenal factors, and its treatment is primarily based on the elimination of these triggering factors (including contrast exposure), supportive care, which includes nutrition, dialysis (with optimal dosing and appropriate indication), prevention of infections, and management of hypertension (HT), electrolyte and acid-base imbalance [5]. The reported risks of CKD transition after AKI are increased age, a lower baseline estimated glomerular filtration rate (eGFR) before the AKI episode, the presence of congestive heart failure (CHF) and HT, the recurrence of AKI, low serum albumin levels during hospitalization, dialysis requirement, and low eGFR levels at hospital discharge [6]. Among these risk factors, our patient had low serum albumin levels during hospitalization, severe AKI (stage 3) with prolonged dialysis need (3 months), and AKI recurrence due to crush syndrome followed by sepsis (Figure 3). Although the renal recovery rate was reported as 42.4% among moderate and severe sepsis patients without trauma complicated with stage 2 and 3 AKI, the degree of recovery in kidney functions and duration of AKI were not mentioned in detail in that study, and moderate AKI patients without the need for KRT were also included in the results. The occurrence of AKI after blunt trauma among 17.341 patients was found as 0.8% and the mortality of these patients was reported as 65% [7].

However, the percentages of patients with high ISS, severe sepsis, and severe AKI with 90 days of HD requirement as in our patient were not presented in that study. The mortality was 65.8% in another study involving severe trauma patients with AKI [8]. It is well-known that severe trauma, leading to severe sepsis and severe AKI, has catastrophic effects on the survival of patients. In addition to polytrauma and sepsis, nephrotoxic antibiotics and contrast media had to be given to our patient, the risk score for prediction of contrast-induced AKI was 14% by then. Colistin and teicoplanin, which lead to further nephrotoxicity in this case, were reported to result in AKI in 39.2-51% and 10.6% of the patients respectively [9]. Despite severe AKI, severe sepsis, and all of the nephrotoxic medications and surgical interventions; our patient had complete renal recovery after 3 months of dialysis dependency. The factors determining renal outcomes among individuals with equal severity of kidney injury may depend on physiological reserve. This reserve is thought to be larger among young patients without any comorbidity as in the present case. The microRNAs, monocyte chemoattractant protein-1, Dickkopf-related protein 3, cell cycle arrest molecules, and myo-inositol oxygenase were indicated to be linked to the CKD transition of AKI patients [10]. We could not have searched these parameters of our patient during his hospitalization for AKI. This is the limitation of this case report. The strengths of this case report are the presentation of the possibility of the complete recovery of the repetitive kidney injuries with catastrophic comorbidities and the importance of optimum interventions of all the comorbidities on the regain of kidney functions.

## Conclusion

A multidisciplinary (including nursery care) approach, early and proper treatment of sepsis, pulmonary rehabilitation, enteral/parenteral nutritional support, and appropriate timing, prescription and dose of dialysis are fundamental factors playing a major role in the recovery of AKI.
